# Detection of Lipid-Rich Prostate Circulating Tumour Cells with Coherent Anti-Stokes Raman Scattering Microscopy

**DOI:** 10.1186/1471-2407-12-540

**Published:** 2012-11-21

**Authors:** Ranjana Mitra, Olivia Chao, Yasuyo Urasaki, Oscar B Goodman, Thuc T Le

**Affiliations:** 1 Nevada Cancer Institute, One Breakthrough Way, Las Vegas, NV, 89135, USA; 2 Roseman University of Health Sciences, 11 Sunset Way, Henderson, NV, 89014, USA; 3 Desert Research Institute, 10530 Discovery Drive, Las Vegas, NV, 89135, USA

## Abstract

**Background:**

Circulating tumour cells (CTC) are an important indicator of metastasis and associated with a poor prognosis. Detection sensitivity and specificity of CTC in the peripheral blood of metastatic cancer patient remain a technical challenge.

**Methods:**

Coherent anti-Stokes Raman scattering (CARS) microscopy was employed to examine the lipid content of CTC isolated from the peripheral blood of metastatic prostate cancer patients. CARS microscopy was also employed to evaluate lipid uptake and mobilization kinetics of a metastatic human prostate cancer cell line.

**Results:**

One hundred CTC from eight metastatic prostate cancer patients exhibited strong CARS signal which arose from intracellular lipid. In contrast, leukocytes exhibited weak CARS signal which arose mostly from cellular membrane. On average, CARS signal intensity of prostate CTC was 7-fold higher than that of leukocytes (P<0.0000001). When incubated with human plasma, C4-2 metastatic human prostate cancer cells exhibited rapid lipid uptake kinetics and slow lipid mobilization kinetics. Higher expression of lipid transport proteins in C4-2 cells compared to non-transformed RWPE-1 and non-malignant BPH-1 prostate epithelial cells further indicated strong affinity for lipid of metastatic prostate cancer cells.

**Conclusions:**

Intracellular lipid could serve as a biomarker for prostate CTC which could be sensitively detected with CARS microscopy in a label-free manner. Strong affinity for lipid by metastatic prostate cancer cells could be used to improve detection sensitivity and therapeutic targeting of prostate CTC.

## Background

Circulating tumour cells (CTC) are malignant cells shed into the bloodstream. These cells spread to distant sites where they initiate metastases, the primary cause of cancer-specific mortality [1]. Patients with metastatic cancers are more likely to have CTC detected in their blood [2]. Hence, CTC are an important indicator of metastasis and are associated with a poor prognosis [3]. Enumeration of CTC is routinely used to ascertain the prognosis and monitor response to cancer treatments [4]. Importantly, CTC represent an instantaneous sampling of the tumour burden and thus hold the key to understanding the stages of cancer metastasis including extracellular matrix degradation, tissue invasion, and escape into the bloodstream [5].

Recognizing the significance of CTC, researchers have devised a number of strategies for CTC detection [4,6]. Four main strategies have been employed. The first strategy focuses on capturing CTC from the blood using either microposts or magnetic beads coated with antibodies against epithelial cell adhesion molecule (EpCAM) which is present in CTC of epithelial origin but absence in leukocytes [7,8]. The second strategy exploits the physical difference in size and density between CTC and leukocytes to separate CTC using size exclusion filtration or density gradient centrifugation [9-12]. The third strategy stains CTC of blood smears using fluorescently conjugated antibodies and scans for CTC using microscope-based cytometry [13]. The fourth strategy detects CTC indirectly by measuring the DNA shed by CTC into the blood using polymerase chain reaction amplification techniques [14]. Another alternative strategy uses initial enrichment with EpCAM molecule followed by detection with tumour specific RT-PCR [15,16].

There are significant deficiencies with current technologies for CTC detection. While EpCAM is expressed in most epithelial cells, the expression level varies from cell to cell [17]. Furthermore, loss of EpCAM expression seen in de-differentiated states such as epithelial-mesenchymal transition and in cancer stem cells, in which EpCAM expression may be undetectable [18,19]. Therefore, EpCAM antibodies-based enrichment methods are inadequate for the detection of CTC populations with heterogeneous EpCAM expression levels. Second, the heterogeneity in size, shape, and density of CTC poses a significant challenge to their isolation based on physical properties [20]. Third, intrinsic fluorescence arising from nicotinamide adenine dinucleotide (NADH) coenzymes of blood cells can interfere with the signal arising from fluorescently-labeled CTC [21]. Fourth, confirmatory staining for the cytoplasmic markers such as cytokeratin requires cell fixation and does not allow recovery of live prostate CTC for further studies [6]. Clearly, new technologies are needed to improve the detection of CTC.

In this paper, we report the detection of lipid-rich CTC in the peripheral blood of metastatic prostate cancer patients with coherent anti-Stokes Raman scattering (CARS) microscopy. CARS microscopy is a label-free and non-perturbative imaging technique highly sensitive to the visualization of lipid-rich structures. We examine intracellular lipid as a potential biomarker of CTC and explore the use of CARS microscopy as a new technology for the detection of lipid-rich CTC.

## Methods

### Blood sample collection and preparation

All experiments involving human subjects were performed with the approval of the University Medical Center of Southern Nevada Institutional Review Board (FWA#0002738 and IRB#NVCI11-15) and were in compliance with the World Medical Association Declaration of Helsinki Declaration on Ethical Principles for Medical Research Involving Human Subjects. Written informed consent was obtained from patients prior to participation in this study. Peripheral blood from the metastatic prostate cancer patients were collected in heparin containing tubes. Metastatic cancer patients were randomly selected for participation in this study with no bias in regard to blood chemistry or clinical characteristics. Blood of sex-age matched healthy volunteers were collected for control. Approximately 7.5 ml of blood per patient or volunteer was transferred into vacutainers containing Ficoll/Hypaque solution for the collection of the buffy coats with centrifugation. After immuno-staining, nucleated cells of the buffy coats were transferred directly onto coverslip-bottom culture dishes and examined with widefield fluorescence microscopy or CARS microscopy. Alternatively, to improve the detection of CTC, the buffy coats were passed through columns containing microbeads conjugated to monoclonal anti-human CD45 antibodies (Cat. No. 130-045-801, Miltenyi Biotec, Auburn, CA) to remove CD45-positive cells. The flow through fractions were collected, immuno-stained and transferred onto coverslip-bottom culture dishes for examination with widefield fluorescence microscopy or CARS microscopy.

### Immunofluorescent staining

Cells of the buffy coats were stained with primary antibodies against CD45 (Cat. No. ab10559, Abcam, Cambridge, MA) and Alexa Fluor 488-conjugated secondary antibodies, and with primary antibodies against cytokeratin (CK, Cat. No. 8018, Santa Cruz Biotechnology, Santa Cruz, CA) and TRITC-conjugated secondary antibodies. The nucleus was stained with Hoeschst 33342 (Cat. No. H21492, Molecular Probes, Eugene, OR). Immuno-staining was performed according to the manufacturers’ recommended protocols.

### CARS microscopy and integrated Raman microspectroscopy

The experimental setup of our home-built CARS microscope has been described previously [22]. The Raman frequency used for the detection of lipid was fixed at 2851 cm^-1^. Bandpass filters for Alexa Fluor 488, TRITC, and CARS, were 510/42 nm, 579/34 nm, and 736/128 nm, respectively. Images were acquired at 10 seconds per frame and presented as 3-D stacks of approximately 20 frames taken at 1-micron increment along the vertical axis. Image analysis of CARS intensity was performed post-acquisition using NIH ImageJ software. Raw CARS average pixel intensity (0-255) of individual cells was analyzed and used for quantitative analysis.

### Cell lines and culture conditions

The non-transformed prostate epithelial cell line RWPE-1 (Cat. No. CRL-11609, ATCC, Manassas, Virginia) was grown in the recommended keratinocyte serum free media. The cell lines BPH-1 and C4-2 were a gift from Dr. Simon Hayward (Vanderbilt University) and Dr. David Nanus (Cornell University), respectively, and were both grown in RPMI media supplemented with 10% FBS. To evaluate lipid uptake with Oil Red O staining, cells were incubated in 50% human plasma for 24 hours at 37^o^C and 5% CO_2_. To track lipid uptake with CARS imaging and Raman microspectroscopy, C4-2 cells were incubated with 50% human plasma spiked with 50 μM of palmitic acid-d31 (Cat. No. 366897, Sigma-Aldrich, St. Louis, MO) for 24 hours at 37^o^C and 5% CO_2_.

### RT-PCR gene expression profiling of fatty acid binding and transport proteins

For RT-PCR profiling, total RNA extracts from cultured cell lines were isolated using RNeasy kit (Qiagen Sample and Assay Technologies, Valencia, CA), the DNase treatment was performed on the column using the recommended protocol. The cDNA was prepared with 1 μg of starting RNA using the RT^2^ First Strand Kit (Qiagen, CA). The RT^2^ SYBR Green ROX qPCR Mastermix (Qiagen, CA) was used for q-PCR reactions and manufactures protocol was followed. The human fatty acid metabolism array (PAHS007) from SA Biosciences (Qiagen, CA) was used for the gene expression profiling of fatty acid binding and transport proteins. RT-PCR data were analyzed using the SABiosciences online analysis software.

## Results and discussion

### Detection of CTC in metastatic prostate cancer patients

**Figure 1:**
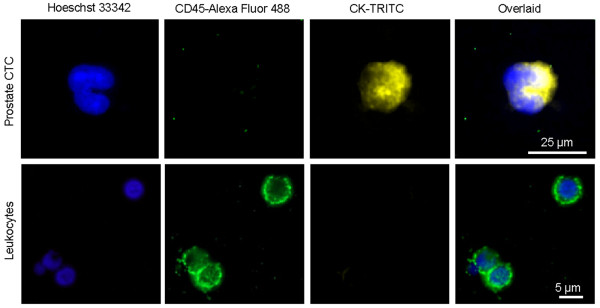
**Detection of circulating tumour cells in the peripheral blood of metastatic prostate cancer patients.** Upper row: CTC were identified as Hoechst33342^+^CD45^-^CK^+^ cells. Lower row: leukocytes were identified as Hoechst33342^+^CD45^+^CK^-^ cells. Images were taken with widefield fluorescent microscopy

In the peripheral blood of metastatic prostate cancer patients, we detected the present of Hoeschst 33342^+^CD45^-^CK^+^ cells (Figure 1, **upper row**), which were absent in the peripheral blood of healthy volunteers. These cells could be clearly distinguished from leukocytes, which were Hoeschst 33342^+^CD45^+^CK^-^ (Figure 1, **lower row**). Based on previous description of CTC in the literature, CD45^-^CK^+^ cells were identified as CTC [6].

### Lipid-rich prostate CTC

**Figure 2:**
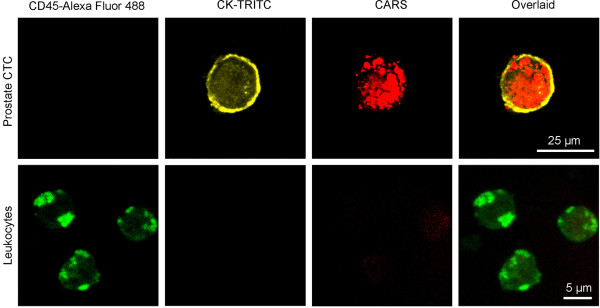
**Detection of lipid-rich prostate CTC with CARS microscopy.** Upper row: Prostate CTC exhibited strong CARS signal arising from intracellular lipid accumulation. Lower row: Leukocytes exhibited weak CARS signal arising mainly from cellular membrane.

**Figure 3:**
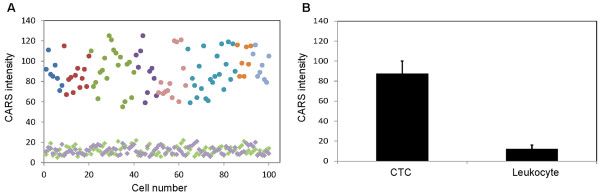
**Average CARS signal intensity of prostate CTC versus leukocytes.** (**A**) CARS intensity, average pixel intensity (0-255) of individual cells, as a function of 100 prostate CTC (circles) and 100 leukocytes (purple squares) in eight metastatic prostate cancer patients and 100 leukocytes from healthy volunteers (green squares). CTC from each individual patient were color-coded (same color circles). (**B**) Average CARS intensity of 100 prostate CTC from cancer patients and 200 leukocytes from both cancer patient and healthy volunteers. Error bars are the standard deviations.

Previously, lipid-rich primary cancer cells and circulating tumour cells in murine cancer model were reported with CARS imaging [23,24]. Here, we examined the lipid content of CD45^-^CK^+^ cells with CARS microscopy. CARS imaging, together with simultaneous two-photon fluorescence (TPF) imaging, revealed that CD45^-^CK^+^ cells exhibited strong cytoplasmic vibrational signal at 2851 cm^-1^, which is a reliable measure of lipid (Figure 2, **upper row**) [25]. In contrast, CD45^+^CK^-^ cells exhibited weak CARS signal at the same vibrational frequency. It is likely that CARS signal arose mainly from cytoplasmic lipid accumulation in CD45^-^CK^+^ cells and from cellular membrane in CD45^+^CK^-^ cells. All 100 CTC identified from the peripheral blood of 8 metastatic prostate cancer patients exhibited strong CARS signal (Figure 3A). Whereas, all leukocytes examined exhibited weak CARS signal. On average, prostate CTC had approximately 7-fold more CARS intensity than leukocytes with a p-value of < 0.0000001 (Figure 3B).

### High affinity for lipid by a metastatic prostate cancer cell line

**Figure 4:**
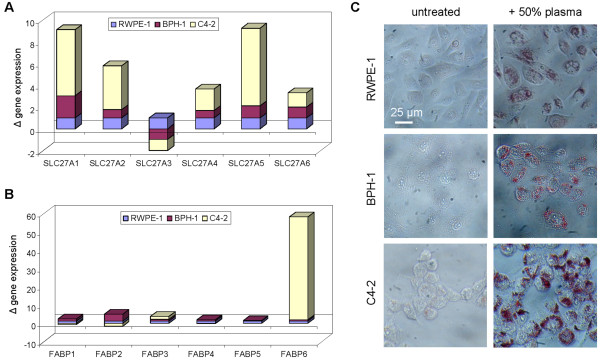
**Gene expression of lipid transport proteins and lipid uptake ability.** Real-time PCR gene expression profiling of (**A**) fatty acid transport proteins (FATPs encoded by SLC27A genes) and (**B**) fatty acid binding proteins (FABPs) of non-transformed prostate epithelial cell line RWPE-1, benign prostatic hyperplasia epithelial cell line BPH-1, and metastatic prostate cancer cell line C4-2. Gene expression levels were normalized to 1 for RWPE-1 cell line and respectively for other cell lines. (**C**) Oil Red O staining to evaluate the uptake of plasma lipid by RWPE-1, BPH-1, and C4-2 cells after 24 hours of incubation with 50% human plasma.

Over-expression of genes encoding for fatty acid metabolism and transport proteins in cancerous versus non-transformed cells was a common observation in many type of cancers including colorectal [26], prostate [27], breast [28], and skin [29] cancers. Indeed, protein-mediated lipid transport accounted for more than 90% of cellular uptake of long-chain fatty acids [30]. To examine the affinity of prostate cancer cells for lipid, we employed real-time PCR to profile for the expression of genes encoding for fatty acid transport proteins (SLC27A1-6) and fatty acid binding proteins (FABP 1-6) in non-transformed prostate epithelial cell line RWPE-1 [31], benign prostatic hyperplasia epithelial cell line BPH-1 [32], and metastatic prostate cancer cell line C4-2 [33,34]. We observed significant increases in the gene expression of 5 out of 6 fatty acid transport proteins (SLC27A1, 2, 4, 5, 6) in C4-2 cells compared to RWPE-1 and BPH-1 cells (Figure 4A). In addition, we observed a nearly 60-fold increase in the gene expression of FABP-6 in C4-2 cells as compared to RWPE-1 and BPH-1 cells (Figure 4B). Interestingly, C4-2 cells exhibited higher lipid uptake ability than RWPE-1 and BPH-1 cells when incubated with 50% human plasma for 24 hours as indicated by Oil Red O (ORO) staining of intracellular lipid droplets (Figure 4C). Our data revealed an increase in the expression of lipid transport proteins and the affinity for lipid in a metastatic prostate cancer cell line compared to non-transformed or non-malignant prostate epithelial cell lines.

### Lipid uptake kinetics of a metastatic prostate cancer cell line

**Figure 5:**
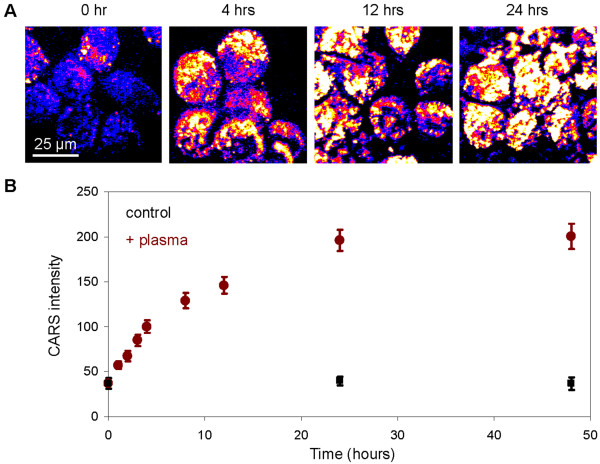
**Plasma lipid uptake kinetics of C4-2 metastatic prostate cancer cells.** (**A**) CARS images of C4-2 cells incubated with 50% human plasma as a function of time. (**B**) CARS intensity of individual C4-2 cells as a function of incubation time with 50% human plasma (red circles) versus control C4-2 cells in growth media (black squares). Error bars represent the standard deviations across 50 cells analyzed per time point.

**Figure 6:**
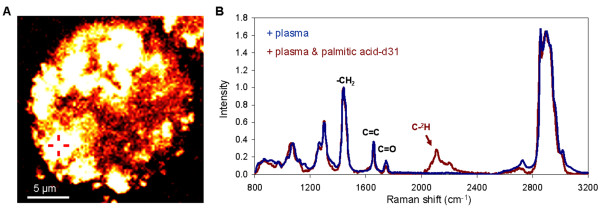
**Tracking the uptake of deuterated palmitic acid with Raman microspectroscopy.** (**A**) CARS image of a C4-2 cell at 24 hours after incubation with 50% human plasma spiked with 50 μM of palmitic acid d-31. Cross-hair indicates a representative location for Raman microspectroscopy analysis. (**B**) Representative of Raman signatures of C4-2 cells incubated in 50% human plasma (blue) or in 50% human plasma spiked with deuterated palmitic acid d-31 (red) for 24 hours.

Taking advantage of the label-free and non-perturbative imaging capability of CARS microscopy, we examined the kinetics of lipid uptake of C4-2 cells following their incubation with 50% human plasma (Figure 5A). We found that C4-2 cells rapidly accumulated intracellular lipid, reaching a plateau at approximately 24 hours post incubation (Figure 6A). To confirm lipid uptake by C4-2 cells, we spiked human plasma with 50 μM of deuterated palmitic acid and traced it with Raman microspectrometry. We found a distinctive carbon-deuterium vibration peak around 2150 cm^-1^ arising from the intracellular lipid of C4-2 cells (Figure 6B). Our data showed that exogenous sources of lipid indeed contributed to the rapid increase in intracellular lipid of C4-2 cells incubated with human plasma.

### Lipid mobilization kinetics of a metastatic prostate cancer cell line

**Figure 7:**
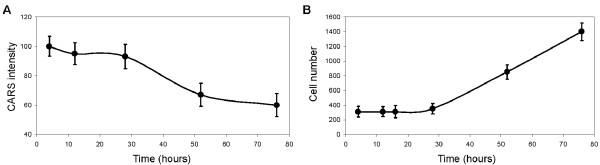
**Lipid mobilization kinetics of C4-2 metastatic prostate cancer cells.** (**A**) CARS intensity of individual C4-2 cells as a function of time. C4-2 cells were first incubated with 50% human plasma for 4 hours, then plasma was removed and replaced with growth media. Error bars represent the standard deviations across 50 cells analyzed per time point. (**B**) Average C4-2 cell number per analysis area of 3 mm^2^ as a function of time after the removal of human plasma. Error bars are standard deviations across 10 areas evaluated at each time point.

Next, we examined the ability of C4-2 cells to mobilize intracellular lipid following the removal of human plasma. We first incubated C4-2 cells in 50% human plasma for 4 hours, then replaced human plasma with cell culture media and monitored intracellular lipid content with CARS microscopy. We found that C4-2 cells had lost only 10% of their intracellular lipid at up to 24 hours after the removal of human plasma (Figure 7A). Compare with lipid uptake kinetics, where 100% of lipid uptake capability was reached at 24 hours post incubation with human plasma (Figure 5B), lipid mobilization kinetics of C4-2 cells occurred at a much slower rate. Between 24 hours and 28 hours post plasma removal, we found a substantial drop in intracellular lipid level of C4-2 cells of nearly 50% (Figure 7A). However, this drop in intracellular lipid level coincided with a doubling in C4-2 cell number (Figure 7B). Thus, the drop in intracellular lipid level of C4-2 cells was most likely a dilution due to cell division. Taken together, our data showed rapid lipid uptake kinetics and slow lipid mobilization kinetics by C4-2 cells.

## Conclusions

### Intracellular lipid as a potential biomarker for CTC

We found that all CTC identified from the peripheral blood of metastatic prostate cancer patients were lipid-rich. Thus, intracellular lipid can potentially serve as an additional biomarker for prostate CTC. Increased expression of lipid metabolism and lipid transport proteins are commonly observed in many types of cancers [27-29]. Therefore, we anticipate that intracellular lipid accumulation is not a property unique to CTC derived from prostate cancer, but a general property for other cancers as well. Indeed, we found that CTC isolated from metastatic bladder, breast, and skin cancer patients were also lipid rich (data not shown). In addition, intracellular lipid accumulation in non-adipocyte cells is a general defence mechanism against exposure to excess extracellular lipid environment [24,35,36]. Therefore, we anticipate that all CTC, and not just a specific CTC subpopulation, to exhibit intracellular lipid accumulation. Because CARS microscopy is a label-free imaging technique highly sensitive to lipid visualization [25], it has the potential for non-perturbative detection of CTC based on their intracellular lipid content. However, given the non-specificity of lipid as a biomarker, it is advisable that lipid be used in conjunction with other established specific fluorescent biomarkers to improve CTC detection sensitivity and accuracy. Fortunately, simultaneous CARS and multicolor TPF imaging can be deployed on any typical CARS microscope [37]. This multimodal imaging capability renders CARS microscopy an attractive means for the detection of CTC. As demonstrated with C4-2 cancer cells, the quantity of intracellular lipid is a function of exposure time to extracellular lipid. It is plausible that CARS signal of CTC intracellular lipid could be used as an indicator of circulation time.

### Exploring the affinity for lipid for improved detection of CTC and delivery of anti-cancer drugs

^11^C-choline is commonly used in the clinic as a radiotracer for positron emission tomography imaging of prostate tumour due to the high affinity of prostate cancer cells for bile acids [38]. The ability of cancer cells to uptake natural fatty acids has also been exploited to deliver anti-cancer drugs. Paclitaxel conjugated to docosahexaenoic acid, or DHA-paclitaxel, has been shown to target lung tumour implants in mice more efficiently with increased delivery kinetics and prolonged retention time [39]. DHA-paclitaxel, also known as Taxolprexin, is currently undergoing clinical trial for the treatment of oesophago-gastric cancer [40]. Similarly, 5-azacytidine conjugated to elaidic fatty acid, or compound CP-4200, has been shown to have higher antitumour activity than unconjugated azacytidine in an orthotopic mouse tumour model for acute lymphocytic leukemia [41]. Encapsulation of anti-cancer drugs in lipid emulsions has also been shown to improve safety and delivery efficiency to lung tumours in murine xenograft models [42,43]. In agreement with the literature, we found strong affinity for plasma lipid by C4-2 metastatic prostate cancer cells *in vitro*. The detection of lipid-rich prostate CTC in metastatic cancer patients suggested a conserved affinity for lipid by metastatic prostate cancer cells *in vivo*. Thus, targeting CTC with fluorescently-conjugated fatty acids or lipophillic anti-cancer drugs should increase the detection sensitivity and therapeutic efficacy, respectively.

### Other lipid-rich blood cells

In the current study, we have not observed any accumulation of intracellular lipid droplets in leukocytes. However, from the literature, monocytes were reported to uptake fatty acids from very low-density lipoprotein lipolysis products and formed small intracellular lipid droplets [44-46]. The lipid contents of monocytes were low overall and declined postprandially [45]. Consistent with our observation, Wu *et al* reported that CD11c^+^ murine wildtype monocytes lacked intracellular lipid. However, CD11c^+^ monocytes from hypercholesterolemic apoE^-/-^ knockout mice contained lipid droplets [47]. Thus, monocytes isolated from human subjects during the peak postprandial period may also contain lipid droplets. It is plausible that lipid accumulation in monocytes could be pronounced following a high-fat meal, particularly in patients with dyslipidemia. Fortunately, lipid-rich monocytes are CD45^+^ cells that can be readily distinguished from CD45^-^ CTC. In addition, CD45^+^ cells can be removed via the use of columns containing microbeads conjugated to monoclonal anti-human CD45 antibodies. Because labeling of CD45 surface marker can be achieved without cell fixation, simultaneous CARS and two-photon fluorescent imaging can be used for the detection of live CTC base on positive CARS signal and negative CD45 fluorescent signal, respectively.

### Improving CTC detection throughput and sensitivity

A significant challenge to any CTC detection method is the ability to enumerate very low number of CTC in the blood. To achieve this goal, the detection method must permit sampling of all individual blood cells. Currently, the most common method for clinical CTC detection relies on immuno-magnetic bead capturing system and microscope-based cytometry [7]. In this paper, we also used immuno-magnetic beads to reject CD45^+^ cell population and enrich CTC population for subsequent CARS imaging. However, immune-magnetic bead system is susceptible to sample loss due to multi-step sample preparation. Microscope-based cytometry requires all cells to be immobilized to the glass surface for laser scanning. This requirement is normally destructive to cells due to high shear stress treatments. In addition, the mechanical motions associated with microscope-stage movements or raster scanning mirrors movements hinder the speed of detection. An alternative to immuno-magnetic beads and microscope-based cytometry has been described using microfluidic flow cytometry with a flow rate of 1-2 ml per hour [8]. Microfluidic flow cytometry generally permits higher sampling rate, less shear stress to cells, and less sample preparation steps than microscope-based cytometry. Previously, microfluidic CARS flow cytometry has been employed for the enumeration of lipid-rich cells and particles [48]. It is conceivable that the deployment of microfluidic CARS/TPF flow cytometry to enumerate CTC will improve both CTC detection throughput and sensitivity. High detection thoughput and large sampling volume associated with microfluidic flow cytometry should eliminate the need for CTC enrichment from patient blood samples.

### Potential for intravital detection of CTC

In recent years, nonlinear optical (NLO) microscopy has been increasingly employed for cancer imaging [24,49-53]. Advances in NLO endoscopy development suggest its potential deployment in cancer centers [54-57]. Most notably is the development of vibrational photoacoustic (VPA) microscopy with millimeter-scale penetration depth that could permit label-free and bond-selective imaging of subcutaneous microvasculature [58]. The discovery of lipid-rich prostate CTC described in this paper suggests possible deployment of VPA together with other NLO imaging modalities for intravital flow cytometry to enumerate CTC in a non-invasive manner without drawing blood [59,60].

## Abbreviations

CTC: Circulation tumour cells; CARS: Coherent anti-Stokes Raman scattering; CK: Cytokeratin; EpCAM: Epithelial cell adhesion molecule; FABPs: Fatty acid binding proteins; FATPs: Fatty acid transport proteins; NLO: Nonlinear optics; VPA: Vibrational photoacoustic.

## Competing interests

A patent has been filed by the Nevada Cancer Institute on behalf of the authors on the methods to detect and isolate CTC.

## Authors’ contributions

RM, OBG, and TTL designed experiments. RM, OC, YU, and TTL performed experiments and analyzed data. RM, OBG, and TTL prepared the manuscript. All authors read and approved final manuscript.

## Pre-publication history

The pre-publication history for this paper can be accessed here:

http://www.biomedcentral.com/1471-2407/12/540/prepub
